# Markers of endothelial and epithelial pulmonary injury in mechanically ventilated COVID-19 ICU patients

**DOI:** 10.1186/s13054-021-03499-4

**Published:** 2021-02-19

**Authors:** Savino Spadaro, Alberto Fogagnolo, Gianluca Campo, Ottavio Zucchetti, Marco Verri, Irene Ottaviani, Tanushree Tunstall, Salvatore Grasso, Valentina Scaramuzzo, Francesco Murgolo, Elisabetta Marangoni, Francesco Vieceli Dalla Sega, Francesca Fortini, Rita Pavasini, Paola Rizzo, Roberto Ferrari, Alberto Papi, Carlo Alberto Volta, Marco Contoli

**Affiliations:** 1 Intensive Care Unit, Department of Translational medicine and for Romagna, University of Ferrara, Azienda Ospedaliera Universitaria di Ferrara, Via Aldo Moro 8, 44124 Ferrara, Italy; 2 Cardiovascular Institute, Azienda Ospedaliero-Universitaria di Ferrara, Cona, FE Italy; 3 Department of Infection Biology, School of Hygiene and Tropical Medicine, Keppel Street, London, WC1E 7HT UK; 4 Dipartimento dell’Emergenza e Trapianti d’Organo (DETO), Sezione di Anestesiologia e Rianimazione, Università degli Studi di Bari “Aldo Moro”, Bari, Italy; 5 Maria Cecilia Hospital, GVM Care and Research, Cotignola, RA Italy; 6 Department of Morphology, Surgery, and Experimental Medicine, Laboratory for Technologies of Advanced Therapies, University of Ferrara, Ferrara, Italy; 7 Respiratory Section, Department of Morphology, Surgery, and Experimental Medicine, University of Ferrara, Ferrara, Italy

**Keywords:** COVID-19, Acute respiratory distress syndrome, Biomarkers, Angiopoietin-2, Intercellular adhesion molecule-1, Vascular cell adhesion protein 1, Receptor for advanced glycation end-products, Selectin

## Abstract

**Background:**

Biomarkers can be used to detect the presence of endothelial and/or alveolar epithelial injuries in case of ARDS. Angiopoietin-2 (Ang-2), soluble intercellular adhesion molecule-1 (ICAM-1), vascular cell adhesion protein-1 (VCAM-1), P-selectin and E-selectin are biomarkers of endothelial injury, whereas the receptor for advanced glycation end-products (RAGE) reflects alveolar epithelial injury. The aims of this study were to evaluate whether the plasma concentration of the above-mentioned biomarkers was different 1) in survivors and non-survivors of COVID-19-related ARDS and 2) in COVID-19-related and classical ARDS.

**Methods:**

This prospective study was performed in two COVID-19-dedicated Intensive Care Units (ICU) and one non-COVID-19 ICU at Ferrara University Hospital. A cohort of 31 mechanically ventilated patients with COVID-19 ARDS and a cohort of 11 patients with classical ARDS were enrolled. Ang-2, ICAM-1, VCAM-1, P-selectin, E-selectin and RAGE were determined with a bead-based multiplex immunoassay at three time points: inclusion in the study (T1), after 7 ± 2 days (T2) and 14 ± 2 days (T3). The primary outcome was to evaluate the plasma trend of the biomarker levels in survivors and non-survivors. The secondary outcome was to evaluate the differences in respiratory mechanics variables and gas exchanges between survivors and non-survivors. Furthermore, we compared the plasma levels of the biomarkers at T1 in patients with COVID-19-related ARDS and classical ARDS.

**Results:**

In COVID-19-related ARDS, the plasma levels of Ang-2 and ICAM-1 at T1 were statistically higher in non-survivors than survivors, (p = 0.04 and p = 0.03, respectively), whereas those of P-selectin, E-selectin and RAGE did not differ. Ang-2 and ICAM-1 at T1 were predictors of mortality (AUROC 0.650 and 0.717, respectively). At T1, RAGE and P-selectin levels were higher in classical ARDS than in COVID-19-related ARDS. Ang-2, ICAM-1 and E-selectin were lower in classical ARDS than in COVID-19-related ARDS (all p < 0.001).

**Conclusions:**

COVID-19 ARDS is characterized by an early pulmonary endothelial injury, as detected by Ang-2 and ICAM-1. COVID-19 ARDS and classical ARDS exhibited a different expression of biomarkers, suggesting different pathological pathways.

Trial registration

NCT04343053**, Date of registration:** April 13, 2020

## Background

SARS-CoV-2 infection can be complicated by the development of an acute respiratory distress syndrome (COVID-19 ARDS) associated with high mortality rate. The severity of the lung injury often requires mechanical ventilation [1–3], and recently, some morphological pathways of the COVID-19-related ARDS have been elucidated in a series of autopsies. The histologic analysis of pulmonary vessels showed widespread thrombosis with microangiopathy [4], diffuse alveolar damage, capillary congestion, necrosis of pneumocytes, interstitial and intra-alveolar edema and platelet–fibrin thrombi [5]. These results suggest that the profound hypoxemia that these patients might experience can be due to both epithelial and endothelial injury. Nonetheless, an in vivo description of the evolution of the disease is still lacking. Biomarkers evaluation can help to understand COVID-19 pathogenesis over-time. This approach may have clinical implications, helping to clarify the characteristics of this peculiar ARDS and, further, enhancing the chances of treatments of this disease.

Angiopoietin-2 (Ang-2), soluble intercellular adhesion molecule-1 (ICAM-1), soluble vascular cell adhesion molecule-1 (VCAM-1), P-selectin, E-selectin are used as biomarkers of endothelium injury, whereas receptor for advanced glycation end-products (RAGE) is considered a marker for alveolar epithelial injury [6]. Previous studies showed the usefulness of these biomarkers in predicting worse outcomes in patients with “classical” ARDS, including mortality [7–9]. Unfortunately, besides studies focused on markers of systemic endothelial dysfunction, such as D-dimers [10], few data are available on other biomarkers in COVID-19-related ARDS. This is relevant since it is not yet established whether the evaluation of biomarkers used in classical ARDS patients could have a prognostic relevance even in COVID-19 ARDS.

Some authors advocate that significant differences exist between classical and COVID-19-related ARDS, the latter being characterized by higher respiratory system compliance [11, 12] and lower recruitability [13, 14]. On the other hand, other authors do not recognize differences between the two types of ARDS since a large observational study suggests similar pathophysiological features and outcomes [15].

The aim of this study was to evaluate whether the plasma levels of “endothelial” and “alveolar” biomarkers (Ang-2, ICAM-1, VCAM-1, P selectin, E-selectin and RAGE) vary over time between survivors and non-survivors in COVID-19-related ARDS patients. Furthermore, we compared the biomarkers expression in COVID-19-related and classical ARDS.

## Methods

### Study design

The present analysis is based on data from the Pro-thrombotic Status in Patients With SARS-Cov-2 Infection (ATTAC-Co) study (ClinicalTrials.gov Identifier: NCT04343053). The ATTAC-co was a prospective, single-centre study performed at the University Hospital of Ferrara (Italy). The present analysis is specifically designed to investigate the relationship between several biomarkers that are indicators of epithelial and endothelial lung injury in consecutive patients with confirmed COVID-19 who were admitted to COVID-19-dedicated Intensive Care Unit between April and June 2020 and needed mechanical ventilation. A group of patients, admitted in the same period in the non-COVID-dedicated ICU, with similar clinical characteristics in terms of ARDS presentation, but negative for SARS-CoV-2 infection were also included as controls. Mechanical ventilation settings in both groups included constant-flow controlled ventilation, a tidal volume of 6 ml/kg of ideal body weight and the PEEP level titrated to the lowest driving pressure. All patients gave their written informed consent. In case of unconsciousness, the informed consent was signed by their next of kin or legal authorized representative.

### Study population

Inclusion criteria were: (a) age > 18 years; (b) confirmed SARS-CoV-2 infection; (c) need of invasive mechanical ventilation; (d) meeting the Berlin criteria definition for ARDS. Patients were excluded from the study in case of pregnancy or do-not-resuscitate order. SARS-CoV-2 infection was confirmed by reverse transcriptase-polymerase chain reaction assay (Liaison MDX, Diasorin, Saluggia, Italy) from nasopharyngeal swab specimen or tracheal aspirate. Clinical management was in accordance with current guidelines and specific recommendations for the COVID-19 pandemic by Health Authorities and Scientific Societies [16].

### Procedures and blood samples

At ICU admission, clinical and physiological variables were collected: age, sex, body mass index (BMI), Sequential Organ Failure Assessment (SOFA) Score, Simplified Acute Physiology Score (SAPS) II, comorbidities and main laboratory data. Respiratory data collected were: ratio of partial pressure of arterial oxygen to fractional concentration of inspired oxygen (PaO_2_/FiO_2_), partial pressure of carbon dioxide (PaCO_2_), end-inspiratory plateau pressure (assessed performing a 5-s end-inspiratory occlusion), positive end-expiratory pressure (PEEP), tidal volume for predicted body weight (Vt/PBW) and static compliance of the respiratory system calculated as tidal volume/(end inspiratory plateau pressure–total PEEP).

Three different samples of venous blood were collected: at the inclusion in the study (T1, after 1 [1, 2] days from start of MV), after 7 ± 2 days (T2) and 14 ± 2 days (T3). Blood withdrawn was performed from an antecubital vein using a 21-gauge needle. All patients underwent blood sampling in the early morning. The first 2 to 4 mL of blood was discarded. The serum and plasma samples were stored at -80 °C. The plasma levels of Ang-2, ICAM-1, VCAM-1, P-selectin, E-selectin and RAGE, were determined with a bead-based multiplex immunoassay (Luminex, Thermo Fisher Scientific, Waltham, MA, USA). The latter laboratory analyses were performed in the Translational Research Center of the Maria Cecilia Hospital, Cotignola (RA), Italy.

Simultaneously to each blood sample, gas exchanges and respiratory mechanics variables were collected.

### Outcomes

The primary outcome was to evaluate the trend of the biomarker’s plasma levels in survivors and non-survivors COVID-19 ARDS patients. The secondary outcome was the differences in respiratory mechanics variables and gas exchanges between survivors and non-survivors. Furthermore, we compared the biomarkers’ plasma levels at T1 in patients with COVID-19-related ARDS and classical ARDS. Finally, we compared clinical characteristics and plasma levels of the biomarkers at ICU admission between patients with COVID-19-related ARDS and classical ARDS. The dataset of classical ARDS was prospectively registered during the same study period, enrolling all consecutive ARDS patients admitted to a non-COVID-19-dedicated ICU at Ferrara Hospital.

### Statistical analysis

Continuous variables with normal distribution were expressed as mean ± SD. Continuous variables with a non-normal distribution were expressed as median and interquartile range. Normal distribution of the variables was tested with the Kolmogorov–Smirnov test. The variables normally distributed were compared by t test; otherwise the Mann–Whitney U was used. Categorical variables were summarized in terms of numbers and percentages and compared using the two-sided Fisher’s exact test. Differences between measurements were analyzed using repeated-measures ANOVA or two-sample Kolmogorov–Smirnov analysis for data with normal or not normal distribution, respectively. When multiple comparisons were made, p values were adjusted by the Bonferroni post hoc procedure. Receiver operator characteristic (ROC) curves were used to analyze the biomarkers’ ability of to predict 90-day mortality. ROC curve analyses are reported as AUROC, with a 95% confidence interval (95% CI). Due to the unpredictable nature of the COVID-19 outbreak, we were unable to assume an “a priori” sample size; as a convenience sample size, we enrolled all consecutive patients with confirmed COVID-19 who were admitted to two COVID-19-dedicated Intensive Care Unit between April and June 2020.

For all comparisons, a p value of ≤ 0.05 was considered statistically significant. When appropriate, 95% confidence intervals (CIs) were calculated. All analyses were performed with SPSS 25 (IBM, USA).

## Results

### Populations

Thirty-one mechanically ventilated patients with COVID-19-related ARDS were included in the study. Patients were mostly male (26/31, 84%), and the most common comorbidities were hypertension (17/31, 42%) and chronic kidney disease (8/31, 26%). Thirteen patients (43%) were successfully weaned within 28 days, and the mean length of stay in ICU was 31 [25–42] days. Hospital mortality was 35% (11 non-survivors). Non-survivors were older (68 ± 6 vs 61 ± 6; p value = 0.05) than survivors, whereas other baseline characteristics were not significantly different (Table 1).

**Table 1:** Baseline characteristics and values of markers of lung injury in survivor versus non-survivors

Variables	Survivors (n = 20)	Non-survivors (n = 11)	p value
Age, years	61 ± 6	68 ± 6	0.05
Male, sex, no. %	17 (85)	9 (82)	0.59
BMI, Kg/m^2^	27.8 ± 4.1	28.9 ± 3.2	0.41
SAPS II at ICU admission	27 [21–38]	31 [21–37]	0.55
SOFA score at ICU admission	4 [2–5]	5 [3–6]	0.23
Length of ICU stay (days)	31 [25–43]	24 [16–31]	0.04
*Comorbidities*			
Hypertension, no. (%)	9 (45)	8 (73)	0.13
Dyslipidemia, no. (%)	4 (20)	1 (9)	0.40
Former smoker, no. (%)	4 (20)	6 (54)	0.06
Diabetes, no. (%)	4 (20)	1 (9)	0.40
COPD, no. (%)	2 (5)	2 (18)	0.45
Chronic kidney disease, no. (%)	3 (15)	5 (45)	0.08
*Laboratory data at inclusion*			
White blood cells, × 10^3^/L	9.8 [7.5–12.9]	10.2 [8.8–12.7]	0.85
Lymphocytes, × 10^3^/L	965 [640–1167]	680 [560–980]	0.18
Hemoglobin, g/dL	12.1 ± 1.8	13.1 ± 1.1	0.11
Platelets count, × 10^3^/L	286 [265–386]	286 [195–332]	0.43
apTT, seconds	39 ± 5	37 ± 5	0.34
INR	1.1 ± 0.1	1.1 ± 0.1	0.35
Fibrinogen mg/dL	708 [655–888]	786 [532–862]	0.87
D-dimer, mcg/mL	31 [14–42]	36 [19–57]	0.31
IL-6, pg/mL	84 [17–149]	60 [37–145]	0.73
*Respiratory variables at admission*			
PaO_2_/ FiO_2_ ratio	183 [124–264]	116 [81–184]	0.045
PaCO_2_, mmHg	48 [36–56]	48 [41–69]	0.40
V_T_/PDW, mL	6.0 ± 0.5	6.0 ± 0.5	0.96
Driving Pressure, cmH_2_O	9 [8–11]	8 [7–14]	0.79
Compliance Respiratory System	57 [36–78]	60 [51–64]	0.95
Plateau pressure, cmH_2_O	18 [15–21]	21 [18–23]	0.11
PEEP setting (cmH_2_O)	12 [9–12]	10 [8–11]	0.07

Data are reported as number (percentage), mean ± standard deviation or median [interquartile range] as appropriate. BMI: body mass index. COPD: chronic obstructive disease. P value: for the comparison between survivors vs non-survivors cases

At ICU admission, the PaO_2_/FiO_2_ ratio was higher in survivors (183 [126–264]) compared to non-survivors (116 [81–184]); p value = 0.04), while there were no differences in other PaCO_2_ or respiratory mechanics variable (Table 1). D-dimer levels did not differ at ICU admission between survivors and non-survivors (31 [14–42] vs 36 [19–57], p = 0.31), but there was an increase over time in non-survivors (p = 0.006 for two-sample Kolmogorov–Smirnov; Additional file 1).

### Biomarkers

**Fig. 1:**
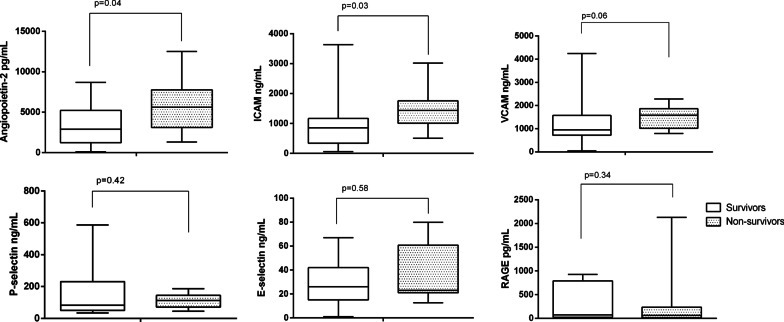
Box-and-whisker plot for comparison of the biomarkers in survivors (n = 20) and non-survivors COVID-19 (n = 11) patients at the inclusion of the study

**Fig. 2:**
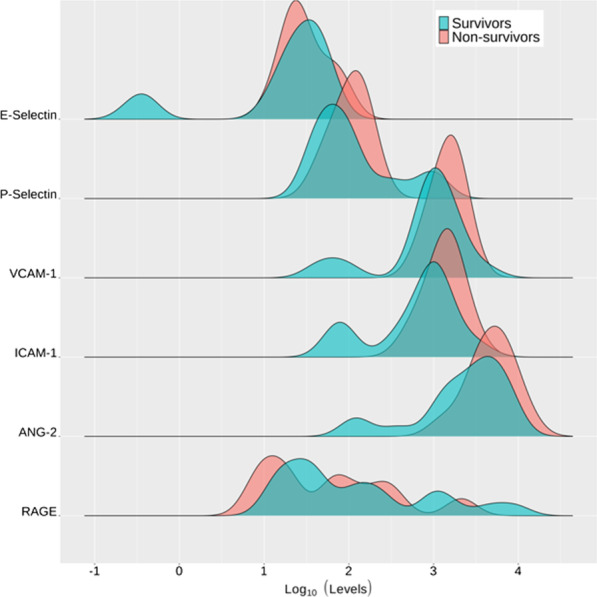
Density distribution of six mediators; RAGE, ANG-2, ICAM-1, VCAM-1, P-Selectin, E-Selectin measured in serum samples collected at timepoint 1. The distribution is colored according to patient outcome, where red relates to patients who died, while blue denotes patients who recovered (survivors, n = 20). The horizontal axis represents the Log_10_ levels of mediators measured in pg/mL for RAGE and ANG-2 and in ng/mL for ICAM-1, VCAM-1, P-Selectin and E-Selectin. The vertical axis corresponds to the respective mediators. Figure generated using R statistical software, version 4.0.2

In COVID-19-related ARDS Ang-2 was higher in non-survivors than in survivors at ICU admission (p = 0.04) and decreased similarly over time in the two groups (p = 0.17 for two-sample Kolmogorov–Smirnov analysis) (Figs. 1, 2, Additional file 2). The area under the receiver operating characteristic curve (AUROC) of Ang-2 at ICU admission for hospital mortality was 0.650. ICAM-1 values were higher in non-survivors than in survivors (p = 0.03 at ICU admission, Fig. 1), and repeated-measure analysis showed more significant decrease from T1 to T3 in survivors compared to non-survivors (p = 0.03 for two-sample Kolmogorov–Smirnov analysis) (Fig. 2, Additional file 2). The AUROC of ICAM-1 for hospital mortality at ICU admission was 0.717. The ICAM-1 plasma level at ICU admission was inversely correlated with the worsening of respiratory system compliance over time (r = -0.470; p = 0.03). VCAM-1 levels at T1 were higher in non-survivors than in survivors, though not statistically significantly (p = 0.06) (Fig. 1). We did not find differences in P-selectin or E-selectin plasma levels at ICU admission or during ICU stay between survivors and non-survivors.

In the overall study population, RAGE decreased significantly during the study period (60.9 [18.8–274.4] at T1, 30.6 [13.4–90.7] at T2 and 20.5 [12.2–41.6] at T3; p value T3 vs T1 < 0.001). RAGE did not differ between survivors and non-survivors at ICU admission (p = 0.34) (Fig. 1, Fig. 2) and had similar decrease overtime (p = 0.71 for two-sample Kolmogorov–Smirnov analysis) (Additional file 2: Table S1).

### Gas exchange and respiratory mechanics

Measures of gas exchange and respiratory mechanics during the study period are reported in Table 2. At ICU admission, the PaO_2_/FiO_2_ ratio was significantly higher in survivors than in non-survivors (p = 0.04), and the PaO_2_/FiO_2_ ratio increased more in survivors than in non-survivors from T1 to T3 (p = 0.001 for two-sample Kolmogorov–Smirnov analysis) (Table 2). PaCO_2_ values did not differ at ICU admission between survivors and non-survivors (p = 0.40), but the PaCO_2_ increased over time more in non-survivors than in survivors (p = 0.001 for two-sample Kolmogorov–Smirnov analysis from T1 to T3). Finally, the respiratory system compliance and the plateau pressure did not significantly differ among the two groups.

**Table 2:** Gas exchange and respiratory mechanics in mechanically ventilated COVID-19 ICU patients

	T1 n = 31	T2 n = 26	T3 n = 20	p value for inter-group trend	p value for group trend comparison
*PaO*_*2*_*/F*_*I*_*O*_*2*_					
Survivor	183 [124–264]	215 [165–304]	251 [204–355]	0.08	0.001
Non-survivor	116 [81–184]	104 [77–147]	144 [94–193]	0.84	
*PaCO*_*2*_					
Survivor	48 [36–56]	45 [38–67]	41 [35–55]	0.22	0.001
Non-survivor	48 [41–69]	63 [50–71]	64 [54–83]	0.02	
*Driving pressure*					
Survivor	9 [8–11]	8 [7–11]	8 [6–8]	0.31	0.10
Non-survivor	8 [7–14]	10 [9–12]	10 [9–12]	0.57	
*Static compliance*					
Survivor	57 [36–78]	71 [41–80]	60 [49–79]	0.07	0.35
Non-survivor	60 [51–64]	41 [24–54]	46 [40–64]	0.23	
*PEEP*					
Survivor	12 [9–12]	8 [6–11]	8 [6–10]	0.36	0.03
Non-survivor	10 [8–11]	14 [9–17]	10 [9–13]	0.93	
*Plateau*					
Survivor	18 [15–21]	17 [14–22]	15 [12–17]	0.25	0.07
Non-survivor	21 [18–23]	22 [19–31]	21 [16–23]	0.84	

Data are reported as median [interquartile range]

### Comparison between COVID-19-related ARDS and classical ARDS

All the biomarkers analyzed differed significantly between COVID-19-related ARDS and classical ARDS at ICU admission (Table 3). In detail, Ang-2, ICAM-1 and E-selectin were higher in COVID-19-related ARDS (all p < 0.001 for group comparison), whereas RAGE and P-selectin levels were higher in classical ARDS. A comparison of clinical characteristics between classical ARDS and COVID-19-related ARDS patients is shown in Additional file 3. Additional data regarding patients with classical ARDS are given in Additional file 4. Patients with classical ARDS had higher hemoglobin and lower D-dimer and international normalized ratio (INR) when compared to COVID-19 patients (Additional file 3). Regarding respiratory mechanics, patients with classical ARDS had higher driving pressure, lower respiratory system compliance, and were ventilated with higher PEEP levels (Additional file 3).

**Table 3:** Comparison of markers of endothelial and epithelial dysfunction between patients with COVID-19-related ARDS and classical ARDS

Biomarker	Covid-19-related ARDS (n = 31)	Classical ARDS (n = 11)	p value
RAGE, pg/mL	60 [18–274]	789 [440–1021]	< 0.001
ICAM-1, ng/mL	1093 [575–1515]	75.7 [63.1–89.6]	< 0.001
VCAM-1, ng/mL	1114 [804–1708]	739 [439–1021]	0.019
Ang-2, pg-mL	3909 [1658–6348]	1045 [627–1654]	< 0.001
P-selectin, ng/mL	93 [50–145]	750 [631–1103]	< 0.001
E-selectin, ng/mL	24.9 [19.2–42.5]	3.3 [2.4–5.7]	< 0.001

Data are reported as median [interquartile range]

### Post hoc analysis

As post hoc analysis, we compared markers of endothelial and epithelial dysfunction in COVID-19 patients with static compliance ≥ 40 mL/cmH_2_O (normal compliance) or < 40 mL/cmH_2_O (low compliance). We were able to show higher values of Ang-2 and E-selectin in patients with normal compliance, and a non-significant trend toward higher RAGE values in patients with low compliance (Additional file 5).

Additionally, due to the observed increasing in PaCO_2_ during ICU stay in non-survivors, we investigated differences in biomarkers at T1 in patients who experienced or not increasing in PaCO_2_. As a result, ICAM-1 and VCAM-1 at T1 were significantly higher in patients who experienced subsequent increased PaCO_2_ (Additional file 6).

Finally, we investigated the ability of the measured biomarkers to identify COVID-19 ARDS rather than classical ARDS. We found that Ang-2 (AUROC 0.75, best cutoff > 2800 pg-mL), RAGE (AUROC 0.69, best cutoff < 208 pg/mL), VCAM-1 (AUROC 0.74, best cutoff > 1312 ng/mL) and ICAM-1 (AUROC 0.63, best cutoff > 1092 ng/mL) were able to discriminate between COVID-19 and classical ARDS.

## Discussion

Our study highlights that a substantial differences exist in COVID-19-related and classical ARDS, as the biomarkers levels of endothelial injury, such as Ang-2 and ICAM-1, were higher in COVID-19-related ARDS, supporting a different pathophysiological pathway of these two syndromes, as recently suggested [11, 17]. Furthermore, we found that some biomarkers of pulmonary endothelial injury, Ang-2 and ICAM-1, are significantly higher in non-survivors patients with COVID-19-related ARDS than in survivors. Conversely, the levels of a biomarker of alveolar epithelial injury, the RAGE, were not different. Our findings suggest that the endothelial injury pathway may be predominant in the pathogenesis of more severe forms of COVID-19-related ARDS.

Previous studies have shown that higher levels of Ang-2 are related to an increased pulmonary vascular leak [18, 19] and that the pulmonary vascular endothelium to up-regulate the ICAM-1 expression in response to inflammation [8, 12]. Other authors found that ICAM-1 can bind alveolar macrophages and enhance inflammatory cytokine production in the alveoli [20]. We found that the plasma levels of Ang-2 and ICAM-1 at T1 were higher in non-survivors than in survivors and, furthermore, i.e., that the plasma levels of Ang-2 and ICAM-1 increased rapidly in COVID-19 non-survivors, within 24 h from ICU admission. To this end, our data might suggest that in COVID-19 ARDS patients the extent of pulmonary endothelium injury, as reflected by ICAM-1 levels, sustains the overall pulmonary inflammation and contribute to the pathogenesis of alveolar epithelial injury. Of note, we found a correlation between ICAM-1 level at admission and worsening of respiratory system compliance during the ICU stay. We thus speculate that evolution of COVID-19-related ARDS toward a more severe phenotype could be related to the extent of pulmonary endothelium injury in the early phase of the disease. Remarkably, the D-dimer levels, a widely used prognostic marker in COVID-19 patients [10, 21, 22]), did not differ between survivors and non-survivors within 24 h from ICU admission.

We were unable to detect any differences in RAGE levels between survivors and non-survivors at ICU admission and throughout the ICU stay. Moreover, the RAGE levels were lower than those previously described in the context of classical ARDS [9]. Since higher RAGE levels have been previously associated with clinical outcomes in patients with classical ARDS, several considerations should be made: *firstly,* alveolar epithelial damage is a less reliably measure when compared to endothelial injury; in this connection, reduced RAGE expression has been reported in patients with idiopathic pulmonary fibrosis, where alveolar epithelial injury is common, and a high prevalence of lung fibrosis is suspected in COVID-19-related ARDS survivors [23]; *secondly,* it has been hypothesized that decreased circulating RAGE could be a marker of deficient inflammatory control [24]. *Finally*, low plasma RAGE was described in respiratory failure due to COPD [25]. It should be noted that RAGE levels may reflect differing mechanisms of lung injury in different lung diseases [24] and hence we can only speculate that the pulmonary endothelial injury is predominant in COVID-19 ARDS, when compared to alveolar injury.

Interestingly, Gattinoni et al. hypothesized that two different COVID-19 ARDS phenotypes can be detected: the first characterized by high compliance and lower recruitability, the second characterized by low compliance and higher recruitability [11]. In a post hoc analysis, we found higher values of Ang-2 and E-selectin in patients with higher compliance. Nonetheless, due to the low number of COVID patients with low compliance, larger cohort studies are warranted to scrutinize the biomarkers associated with different phenotype(s) of COVID-19–related ARDS.

When comparing COVID-19-related ARDS with a cohort of patients with classical ARDS, we observed a significant difference in all the evaluated biomarkers. Ang-2 and ICAM-1 were higher in COVID-19-related ARDS, further highlighting the role of pulmonary vascular injury in this context. On the other hand, RAGE was higher in classical ARDS patients. P-selectin was higher in classical ARDS patients, whereas E-selectin was higher in COVID-19-related ARDS (Table 3). The different behavior of these two-selectin markers further highlights the predominant role of the endothelium, the primary source of E-selectin [26], in the genesis of COVID-19-related ARDS.

This is a hypothesis-generating study, and we must acknowledge some limitations. First, this is a single-centre prospective study focused on the description of pathological alteration in COVID-19-related ARDS. The clinical relevance of these findings should be confirmed in future interventional studies. Secondly, we enrolled patients needing mechanical ventilation and thus our results could not be extended to mild or moderate COVID-19. Third, recent findings suggest that combining respiratory system compliance with D-dimer values can better characterize patients with COVID-19 ARDS; nonetheless, we were unable to perform this analysis due to the fact that almost all our patients (28/31) had D-dimer levels higher than 1880 ng/mL, the cutoff suggested to distinguish between low and high D-dimer [10]. Furthermore, our study investigated multiple biomarkers of endothelial dysfunction and only one marker of epithelial injury. The choice of RAGE as only marker of alveolar epithelial injury, even if consistent with recent studies [27–30], must take into account the intrinsic limits of that biomarker (for example, its ubiquity distribution); additional biomarkers of endothelial injury, such as surfactant protein-D, would have strength our findings.

## Conclusions

The most severe forms of COVID-19 ARDS are characterized by the predominance of the “endothelial” over the “alveolar” injury, as detected by the higher levels of Ang-2 and ICAM-1 in non-survivors compared to survivors; COVID-19 ARDS and classical ARDS had similar loss in gas exchange, but exhibited a different expression of biomarkers, suggesting different pathological pathways.

## Supplementary Information

**Additional file 1.** **Figure S1**: Box-and-whisker plot of D-dimer in survivors(n=20) and non-survivors (n=11) COVID-19 patients during the study period.

**Additional file 2.** **Table S1**: Markers of endothelial and epithelial dysfunction in mechanically ventilated COVID-19 ICU patients.

**Additional file 3.** **Table S2**: Comparison of characteristics at ICU admission between COVID-19-related ARDS and “classical” ARDS patients.

**Additional file 4.** **Table S3**: Individual clinical characteristics and respiratory variables of patients with “classical” ARDS.

**Additional file 5.** **Table S4**: Comparison of markers of endothelial and epithelial dysfunction between COVID-19 patients with static compliance≥40 mL/cmH2O (normal compliance) or <40 mL/cmH2O (low compliance).

**Additional file 6.** **Table S5**: Comparison of markers of endothelial and epithelial dysfunction between COVID-19 patients with increased or non-increased PaCO2.

## Data Availability

The datasets generated during the current study are available from the corresponding author on reasonable request.

## Abbreviations

Ang-2: Angiopoietin-2
ARDS: Acute respiratory distress syndrome
COVID-19: Coronavirus disease 2019
ICAM-1: Soluble intercellular adhesion molecule-1
ICU: Intensive Care Unit
RAGE: Soluble receptor for advanced glycation end-products
SARS-CoV-2: Severe acute respiratory syndrome coronavirus 2
E-selectin: Soluble E-selectin
VCAM-1: Soluble vascular cell adhesion molecule-1
