# Supplementary material for: Markers of endothelial and epithelial pulmonary injury in mechanically ventilated COVID-19 ICU patients
Source: Crit Care. 2021 Feb 19;25:74. doi: 10.1186/s13054-021-03499-4 (PMC7894238; doi:10.1186/s13054-021-03499-4)
Supplement: Supplementary file 2 — Additional file 2. Table S1: Markers of endothelial and epithelial dysfunction in mechanically ventilated COVID-19 ICU patients. [file 13054_2021_3499_MOESM2_ESM.docx]

**Additional file 2. Markers of endothelial and epithelial dysfunction in mechanically ventilated COVID-19 ICU patients.**

|  | **T1** | **T2** | **T3** | **p- value**  **For inter-group trend** | **p value for group trend comparison** |
| --- | --- | --- | --- | --- | --- |
| **Ephitelium** | n=31 | n=26 | n=20 |  |  |
| **RAGE (pg/mL)** | | | | | |
| All | 60.9 [18.8 – 274.4] | 30.6 [13.4 – 90.7] | 20.5 [12.2 – 41.6] | <0.001 |  |
| Survivor | 73.8 [24.0 – 790.1] | 27.2 [9.6 – 38.3] | 18.8 [12.2 – 38.5] | 0.17 | 0.71 |
| Non survivor | 60.9 [12.0 – 237.8] | 54.7 [17.6 – 198.7] | 22.2 [13.9 – 73.5] | 0.19 |
| **Endothelium** |  |  |  |  |  |
| **Ang-2 (pg/mL)** | | | | | |
| All | 3909 [1658 – 6348] | 3310 [2005 – 6381] | 2994 [1709 – 4227] | 0.62 |  |
| Survivor | 2905 [1229 – 5225] | 3033 [1637 – 6366] | 2814 [1639 – 4268] | 0.38 | 0.17 |
| Non survivor | 5634 [3119 – 7774] | 5207 [2060 – 6935] | 3690 [1773 – 4400] | 0.47 |
| **ICAM (ng/mL)** | | | | | |
| All | 1093 [575 – 1515] | 845 [654 – 1461] | 670 [481 – 1095] | 0.14 |  |
| Survivor | 1114 [717 – 1391] | 763 [646 – 1278] | 650 [465 – 897] | 0.11 | 0.03 |
| Non survivor | 1438 [1007 – 1754] | 1440 [590 – 2666] | 1152 [487 – 2640] | 0.31 |
| **P-selectin (ng/ml)** | | | | | |
| All | 92.8 [50.1 – 145.0] | 73.7 [52.8 – 135.8] | 43.9 [18.8 – 196.4] | 0.86 |  |
| Survivor | 83.3 [50.1 – 231.2] | 71.8 [60.7 – 90.3] | 60.7 [42.6 – 130.5] | 0.64 | 0.59 |
| Non survivor | 113.8 [71.2 – 145.1] | 102.8 [40.0 – 168.2] | 102.8 [72.5 – 151.3] | 0.91 |
| **E-selectin (ng/ml)** | | | | | |
| All | 24.9 [19.2 – 42.5] | 34.9 [20.8 – 61.0] | 26.0 [17.8 50.6] | 0.25 |  |
| Survivor | 26.1 [15.4 – 42.4] | 40.3 [22.9 – 64.5] | 24.2 [15.8 – 53.8] | 0.15 | 0.43 |
| Non survivor | 23.1 [23.3 – 60.8] | 32.4 [20.0 – 44.8] | 29.0 [19.9 – 67.7] | 0.25 |
| **VCAM (ng/mL)** | | | | | |
| All | 1114 [804 – 1708] | 1032 [781 – 1818] | 1169 [787 – 1460] | 0.22 | 0.58 |
| Survivor | 984 [720 – 1572] | 930 [716 – 1363] | 956 [726 – 1469] | 0.17 |
| Non survivor | 1584 [1016 – 1866] | 1261 [939 – 1890] | 1251 [1072 – 1497] | 0.35 |

Data are reported as median [interquartile range].
