# Supplementary material for: Markers of endothelial and epithelial pulmonary injury in mechanically ventilated COVID-19 ICU patients
Source: Crit Care. 2021 Feb 19;25:74. doi: 10.1186/s13054-021-03499-4 (PMC7894238; doi:10.1186/s13054-021-03499-4)
Supplement: Supplementary file 3 — Additional file 3. Table S2: Comparison of characteristics at ICU admission between COVID-19-related ARDS and “classical” ARDS patients. [file 13054_2021_3499_MOESM3_ESM.docx]

**Additional file 3. Comparison of characteristics at ICU admission between COVID-19 related ARDS and “classical” ARDS patients.**

| **Variables** | **COVID-19**  **n=31** | **“Classical” ARDS**  **n=10** | **p value** |
| --- | --- | --- | --- |
| Age, years | 64 [57 – 70] | 70 [66 – 80] | 0.038 |
| Male, sex, no. % | 26 (84) | 7 (70) | 0.41 |
| BMI, Kg/m^2^ | 27.7 ± 4.2 | 27.8 ± 4.1 | 0.53 |
| SAPS II at ICU admission | 28 [21 – 37] | 46 [36 – 53] | 0.001 |
| SOFA score at ICU admission | 4 [2 – 5] | 8 [6 – 10] | 0.001 |
| **Laboratory data at inclusion** |  |  |  |
| White blood cells, x10^3^/L | 9.9 [7.8 – 12.9] | 12.1 [8.5 – 15.2] | 0.33 |
| Lymphocytes, x 10^3^/L | 800 [580 – 1080] | 850 [500 – 1950] | 0.64 |
| Haemoglobin, g/dL | 9.9 [9 – 11.2] | 12.1 [11.1 – 13.9] | 0.004 |
| Platelets count, x10^3^/L | 286 [247 – 373] | 253 [159 – 279] | 0.08 |
| apTT, seconds | 39 [36 – 40] | 34 [32 – 39] | 0.07 |
| INR | 1.1 ± 0.1 | 1.05 ± 0.05 | 0.01 |
| Fibrinogenm mg/dL | 726 [617 – 884] | 464 [452 – 804] | 0.11 |
| D-dimer, mcg/mL | 32 [18 – 43] | 8.5 [7 – 20] | 0.007 |
| **Respiratory variables at admission** |  |  |  |
| PaO_2_/ FiO_2_ ratio | 168 [105 – 212] | 188 [101 – 210] | 0.74 |
| PaCO_2_, mmHg | 48 [36 – 59] | 44 [36 - 50] | 0.87 |
| V_T_/PDW, mL | 6.0 ± 0.5 | 6.0 ± 0.5 | 0.99 |
| Driving Pressure, cmH_2_O | 8 [8 – 12] | 13 [10 – 14] | 0.005 |
| Compliance Respiratory System | 58 [42 – 75] | 29 [27 – 37] | <0.001 |
| Plateau pressure, cmH_2_O | 19 [16 – 22] | 26 [22 – 28] | <0.001 |
| PEEP setting (cmH_2_O) | 10 [8 – 12] | 12 [12 – 15] | <0.001 |

Data are reported as number (percentage). mean ± standard deviation or median [interquartile range] as appropriate.
