# Supplementary material for: Markers of endothelial and epithelial pulmonary injury in mechanically ventilated COVID-19 ICU patients
Source: Crit Care. 2021 Feb 19;25:74. doi: 10.1186/s13054-021-03499-4 (PMC7894238; doi:10.1186/s13054-021-03499-4)
Supplement: Supplementary file 4 — Additional file 4. Table S3: Individual clinical characteristics and respiratory variables of patients with “classical” ARDS. [file 13054_2021_3499_MOESM4_ESM.docx]

**Additional file 4. Individual clinical characteristics and respiratory variables of patients with “classical” ARDS.**

| **Patient** | Age, years | ARDS risk factor | Bacterial isolated | PaO_2_/ FiO_2_ ratio | PaCO_2_, mmHg | Driving Pressure, cmH_2_O | PEEP, cmH_2_O | Compliance , mL/cmH_2_O | Prone position |
| --- | --- | --- | --- | --- | --- | --- | --- | --- | --- |
| 1 | 80 | Pneumonia | N/A | 81 | 35 | 12 | 10 | 27 | 0 |
| 2 | 66 | Pneumonia | *P. Aeruginosa* | 210 | 31 | 14 | 12 | 26 | 0 |
| 3 | 85 | Pneumonia | *E. coli* | 229 | 44 | 10 | 12 | 37 | 0 |
| 4 | 40 | Sepsis/Pneumonia | *E. Fecalis* | 188 | 46 | 13 | 15 | 28 | 1 |
| 5 | 69 | Pneumonia | *N/A* | 77 | 55 | 16 | 18 | 24 | 1 |
| 6 | 70 | Pneumonia | *A. Baumanii* | 212 | 61 | 10 | 12 | 38 | 0 |
| 7 | 80 | Sepsis/Pneumonia | *E. Cloacae* | 141 | 50 | 12 | 14 | 35 | 0 |
| 8 | 66 | Pneumonia | *E. Fecalis* | 202 | 44 | 10 | 12 | 39 | 0 |
| 9 | 73 | Sepsis/Pneumonia | *E. Coli* | 175 | 36 | 14 | 14 | 29 | 0 |
| 10 | 69 | Pneumonia | *P. Aeruginosa* | 101 | 45 | 15 | 15 | 28 | 1 |
| 11 | 50 | Pneumonia | *S. Aureus* | 199 | 48 | 14 | 12 | 29 | 0 |
| Total | 70 [66 – 80] |  |  | 188 [101 – 210] | 44 [36 - 50] | 13 [10 – 14] | 12 [12 - 15] | 29 [27 – 37] |  |
