# Supplementary material for: Markers of endothelial and epithelial pulmonary injury in mechanically ventilated COVID-19 ICU patients
Source: Crit Care. 2021 Feb 19;25:74. doi: 10.1186/s13054-021-03499-4 (PMC7894238; doi:10.1186/s13054-021-03499-4)
Supplement: Supplementary file 5 — Additional file 5. Table S4: Comparison of markers of endothelial and epithelial dysfunction between COVID-19 patients with static compliance≥40 mL/cmH2O (normal compliance) or <40 mL/cmH2O (low compliance). [file 13054_2021_3499_MOESM5_ESM.docx]

**Additional file 5. Comparison of markers of endothelial and epithelial dysfunction between COVID-19 patients with static compliance≥40 mL/cmH_2_O (normal compliance) or <40 mL/cmH_2_O (low compliance)**

| Variable | Normal compliance (n=25) | Low compliance (n=6) | p value |
| --- | --- | --- | --- |
| RAGE, pg/mL | 39 [19 – 198] | 611 [49 – 3501] | 0.10 |
| ICAM-1, ng/mL | 1093 [680 – 1534] | 620 [76 – 1560] | 0.34 |
| VCAM-1, ng/mL | 1114 [853 – 1713] | 818 [58 – 2272] | 0.42 |
| Ang-2, pg-mL | 4145 [2844 – 6727] | 903 [128 – 2730] | 0.002 |
| P-selectin, ng/mL | 83 [50 – 138] | 451 [82 – 1045] | 0.06 |
| E-selectin, ng/mL | 30 [22 – 45] | 6 [1 – 21] | 0.001 |

Data are reported as median [interquartile range].
