# Supplementary material for: Markers of endothelial and epithelial pulmonary injury in mechanically ventilated COVID-19 ICU patients
Source: Crit Care. 2021 Feb 19;25:74. doi: 10.1186/s13054-021-03499-4 (PMC7894238; doi:10.1186/s13054-021-03499-4)
Supplement: Supplementary file 6 — Additional file 6. Table S5: Comparison of markers of endothelial and epithelial dysfunction between COVID-19 patients with increased or non-increased PaCO2. [file 13054_2021_3499_MOESM6_ESM.docx]

**Additional file 6. Comparison of markers of endothelial and epithelial dysfunction between COVID-19 patients with increased or non-increased PaCO_2_.**

| Variable | Non-increased PaCO_2_ (n=19) | Increased PaCO_2_ (n=12) | p value |
| --- | --- | --- | --- |
| RAGE, pg/mL | 49 [19 – 1013] | 66 [19 – 209] | 0.70 |
| ICAM-1, ng/mL | 794 [320 – 1161] | 1387 [1106 – 1902] | 0.003 |
| VCAM-1, ng/mL | 946 [697 – 1418] | 1647 [1421 – 1871] | 0.005 |
| Ang-2, pg-mL | 4021 [1163 – 6433] | 3358 [2542 – 561] | 0.73 |
| P-selectin, ng/mL | 93 [49 – 141] | 103 [72 – 156] | 0.41 |
| E-selectin, ng/mL | 27 [19 – 43] | 24 [21 – 56] | 0.86 |

Data are reported as median [interquartile range].
